# Ghrelin Levels in Children With Intestinal Failure Receiving Long-Term Parenteral Nutrition

**DOI:** 10.3389/fnut.2022.896328

**Published:** 2022-05-11

**Authors:** Lotte E. Vlug, Patric J. D. Delhanty, Esther G. Neelis, Martin Huisman, Jenny A. Visser, Edmond H. H. M. Rings, René M. H. Wijnen, Sjoerd C. J. Nagelkerke, Merit M. Tabbers, Jessie M. Hulst, Barbara A. E. de Koning

**Affiliations:** ^1^Division of Gastroenterology, Department of Pediatrics, Erasmus MC Sophia Children’s Hospital, University Medical Center Rotterdam, Rotterdam, Netherlands; ^2^Department of Internal Medicine, Erasmus MC, University Medical Center Rotterdam, Rotterdam, Netherlands; ^3^Division of Gastroenterology, Department of Pediatrics, Willem Alexander Children’s Hospital, Leiden University Medical Center, Leiden, Netherlands; ^4^Department of Pediatric Surgery, Erasmus MC Sophia Children’s Hospital, University Medical Center Rotterdam, Rotterdam, Netherlands; ^5^Division of Gastroenterology, Department of Pediatrics, Emma Children’s Hospital, Amsterdam University Medical Centers, Amsterdam, Netherlands; ^6^Division of Paediatric Gastroenterology, Hepatology and Nutrition, The Hospital for Sick Children, Toronto, ON, Canada

**Keywords:** acylated ghrelin, unacylated ghrelin, short bowel syndrome, enteral autonomy, intestinal adaptation, pediatrics

## Abstract

**Background:**

Children with intestinal failure (IF) require parenteral nutrition (PN). Transition to oral and enteral nutrition (EN) can be difficult also due to abnormal gastrointestinal motility. The gut hormone ghrelin is increased in states of negative energy balance, functioning to preserve euglycemia, and also has appetite stimulating and prokinetic properties. We aimed to evaluate and compare ghrelin levels in children with IF, and to assess the relationship with PN-dependency.

**Methods:**

In this exploratory prospective multicenter study, plasma acylated (AG) and unacylated (UAG) ghrelin levels were measured in children with short bowel syndrome (SBS) and with functional IF (pseudo-obstruction or any enteropathy) and compared with healthy control subjects. Spearman’s rho (r_s_) was used to assess correlations of AG and UAG with PN-dependency (%PN) and parenteral glucose intake.

**Results:**

Sixty-four samples from 36 IF-patients were analyzed. Median baseline AG and UAG levels were respectively 279.2 and 101.0 pg/mL in children with SBS (*n* = 16), 126.4 and 84.5 pg/mL in children with functional IF (*n* = 20) and 82.4 and 157.3 pg/mL in healthy children (*n* = 39). AG levels were higher in children with SBS and functional IF than in healthy children (*p* = 0.002 and *p* = 0.023, respectively). In SBS, AG positively correlated with %PN (r_s_ = 0.5, *p* = 0.005) and parenteral glucose intake (r_s_ = 0.6, *p* = 0.003). These correlations were not observed in functional IF.

**Conclusion:**

Children with IF had raised AG levels which could be related to starvation of the gut. The positive correlation between AG and glucose infusion rate in SBS suggests an altered glucoregulatory function.

## Introduction

Children with intestinal failure (IF) due to short bowel syndrome (SBS), dysmotility or enteropathy have insufficient gut mass or gut function. They suffer from severe malabsorption requiring parenteral nutrition (PN) (1). PN is life-saving for these children, but also associated with complications. Therefore, the ultimate goal in their treatment is to gradually increase enteral nutrition (EN) (i.e., oral feeding and tube feeding) and decrease and eventually stop PN (2). However, increasing EN is often difficult due to suspected gastrointestinal motility problems. Numerous hormones are involved in the regulation of gastrointestinal motility, of which one is ghrelin (3).

Ghrelin is a gut derived peptide hormone, produced predominantly in the fundus of the stomach, but also in lower amounts in the small and large intestine (4). It plays an important role in modulation of growth hormone secretion, it stimulates appetite and food intake and also has prokinetic functions (5, 6). During fasting or starvation, ghrelin levels increase, and its glucoregulatory role becomes apparent (7). Current understanding is that ghrelin’s primary biological role is to maintain blood glucose during such periods of physiological negative energy balance (8). Ghrelin circulates in the blood in acylated (AG) and unacylated (UAG) forms. If blood samples are processed optimally, AG and UAG levels are similar in healthy individuals (9). Under normal conditions, AG levels decrease as the stomach fills following a meal, and increase when the stomach becomes empty after a period of fasting. AG induces a positive energy balance through its appetite stimulating properties, which can lead to weight gain (10). UAG is thought to have protective effects on beta cells and muscle cells and improves glycemic control (11). Because AG and UAG have counterregulatory actions, the AG/UAG ratio is often used to describe their relative effects.

In animal studies using rat models, an increase in ghrelin levels was found after massive small bowel resection (12, 13). There are only a few studies assessing ghrelin levels in patients with IF, and these were only performed in adult patients with SBS (i.e., insufficient gut mass), showing contradictory findings (12, 14). Nothing is known about ghrelin levels in pediatric patients with SBS, or patients with pediatric pseudo-obstruction syndrome or an enteropathy (i.e., insufficient gut function). In the search to gain insight into the underlying mechanisms of intolerance to EN in children with IF, we aimed to explore ghrelin (AG and UAG) levels and its associations with other outcomes in children with SBS and functional IF receiving long-term PN.

## Methods

### Study Design and Participants

The current exploratory analysis was part of a prospective observational cohort study, for which we included children with IF receiving home PN for ≥6 months (indicating severe IF), attending the multidisciplinary IF teams of the Erasmus MC University Medical Center Sophia Children’s Hospital (Rotterdam, Netherlands) and the Amsterdam University Medical Center Emma Children’s Hospital, location AMC (Amsterdam, Netherlands), from March 2015 onward. Dutch healthy control subjects in the same age range, assessed in a study using the same method for AG and UAG measurement, were used as comparisons (15).

The study was conducted in line with the principles of the Declaration of Helsinki and approved by the local research ethical committees (MEC 2015-002, Dutch Trial Register NL5892, https://www.trialregister.nl/trial/5892). Written informed consent was obtained from the caregivers, and, if ≥12 years, the patients themselves.

### Data Collection

Patients were divided into SBS (i.e., insufficient gut mass) and functional IF (i.e., insufficient gut function due to pediatric pseudo-obstruction syndrome or any enteropathy). Clinical and demographic data were retrieved from the medical charts. Data on serum glucose and iron, use of steroids, proton pump inhibitors and antibiotics at the time of sample collection were reported, since these are factors known to influence ghrelin levels (8, 16, 17). Data on nutritional intake (PN and EN) were collected at the time of sample collection.

Parenteral nutrition and EN were prescribed according to our local protocol which is based on the European Society for Paediatric Gastroenterology, Hepatology and Nutrition (ESPGHAN) and European Society for Clinical Nutrition and Metabolism (ESPEN) guideline (18). Glucose infusion did not exceed 18 g/kg body weight per day (≈glucose infusion rate of 13 mg/kg/min) to prevent hyperglycemia, increased lipogenesis and fat tissue deposition together with subsequent liver steatosis. A dietitian of the IF team adjusted the PN and EN prescription guided by routine growth parameters and symptoms of EN intolerance such as vomiting and diarrhea. PN-dependency was defined as percentage of energy intake provided by PN (%PN) = (daily energy intake in kcal provided by PN/total daily energy intake in kcal) × 100.

Body weight and height were measured on the day of blood sample collection. Sex- and age-adjusted standard deviation scores (SDS) were calculated for weight, height, weight-for-height and body mass index (BMI) by using the latest available Dutch national reference standards, with Growth Analyser Research Calculation Tool^1^ (19).

### Procedure

Blood was drawn during standard visits to the outpatient clinic in the afternoon or during a hospital admission. We intended to draw blood after a minimum of 4 h of fasting [i.e., no EN (oral or tube feeding)]. In the healthy control subjects, blood was drawn in the morning after a 12 h overnight fast (15). Blood samples were collected in EDTA tubes and pretreated with 2 mg/ml of 4-(2-aminoethyl)benzenesulfonyl fluoride hydrochloride (AEBSF, Sigma-Aldrich Chemicals) to prevent deacylation of AG and improve ghrelin stability. After centrifugation, plasma was stored at −80°C and assayed within 3 months following collection. A description of the two-step double-antibody sandwich enzyme linked immunosorbent assay (ELISA) for assessment of plasma AG and UAG levels is provided elsewhere (15). We evaluated the first blood sample after March 2015 and, if available, the last blood sample before August 2018.

### Statistical Analysis

For the demographic and clinical patient characteristics, values are expressed as median with 25th to 75th percentile [interquartile range (IQR)] or as a frequency count with percentage. With one-sample Wilcoxon signed rank test, first AG and UAG levels and AG/UAG ratio of patients with SBS and functional IF were compared with a reference population of Dutch healthy control subjects (15). Also, first AG and UAG levels and AG/UAG ratios between patients with SBS and functional IF were compared using Mann–Whitney U tests. Correlations of AG and UAG levels and AG/UAG ratio with %PN and PN glucose intake in g/kg body weight per day were quantified using Spearman’s rho (r_s_). For patients with follow-up AG and UAG measurements, paired samples Wilcoxon tests were used to compare the first and last AG and UAG levels and AG/UAG ratio within patients. A two-tailed *p*-value of <0.05 was considered to indicate statistical significance. Data analyses were performed using Statistical Package for the Social Sciences, Version 25.0 (IBM SPSS Statistics for Windows, Armonk, NY, United States), and GraphPad Prism version 8.00 for Windows (GraphPad Software^2^).

## Results

### Short Bowel Syndrome

#### Patient Characteristics

We included 16 patients with SBS [median age 5.6 years (IQR 3.2–8.7), 5 females]. The most common underlying disease was intestinal atresia (*n* = 6), followed by volvulus (*n* = 4). None of the patients underwent gastric or duodenal resection. At the time of the first AG and UAG measurement, included patients were dependent on PN for a median of 4.4 years (IQR 1.0–6.6) for 61.4% of their nutritional intake (IQR 42.8–100). Patient characteristics are shown in Table 1.

**TABLE 1:** Patient characteristics at the time of the first ghrelin measurement.

		Short bowel syndrome	Functional IF	Healthy control subjects from Kuppens et al. (15)^a^
		*N* = 16	*N* = 20	*N* = 39
		*n* (%) or median [IQR]	*n* (%) or median [IQR]	*n* (%) or median [IQR]
Sex, female		5 (31)	11 (55)	26 (67)
Age, years		5.6 [3.2–8.7]	7.3 [4.1–13.8]	7.3 [3.6–13.6]
Underlying disease				
Intestinal atresia		6 (38)		
Volvulus		4 (25)		
Herniation and strangulation small bowel		2 (13)		
Necrotizing enterocolitis		2 (13)		
Gastroschisis		1 (6)		
Long gap esophageal atresia		1 (5)		
Pediatric intestinal pseudo-obstruction			9 (45)	
Microvillus inclusion disease			3 (15)	
Trichohepatoenteric syndrome			1 (5)	
Protein losing enteropathy			1 (5)	
Long segment Hirschsprung’s disease			1 (5)	
Functional, other			5 (25)	
Remaining small intestinal length, cm	*n* = 15	50 [15–100]		
Ileocecal valve in situ		2 (13)		
Duration of PN-dependency, years		4.4 [1.0–6.6]	6.6 [2.4–13.2]	
Percentage of energy intake provided by PN		61.4 [42.8–100.0]	100.00 [77.4–100.0]	
Energy from PN, kcal/kg/day		53.5 [40.7–74.1]	57.6 [35.7–74.3]	
Glucose in PN, g/kg/day		8.5 [4.4–13.2]	10.0 [6.5–13.9]	
Lipids in PN, g/kg/day		1.3 [1.0–1.9]	1.1 [0.9–1.6]	
Amino acids, g/kg/day		2.2 [1.5–2.9]	2.5 [2.0–3.4]	
Weight-for-age, SDS		−0.7 [−1.4 to 0.2]	−1.0 [−1.8 to −0.4]	−0.2 [−0.8 to 0.5]
Height-for-age, SDS		−1.2 [−1.7 to −0.3]	−1.8 [−2.2 to −1.3]	−0.1 [−0.8 to 0.4]
Weight-for-height, SDS		0.1 [−0.5 to 0.8]	0.3 [−0.5 to 0.8]	
Body mass index, SDS		−0.1 [−0.3 to 0.8]	0.1 [−0.6 to 0.7]	−0.2 [−0.8 to 0.8]
Glucose, mmol/L	*n* = 29	5.2 [4.7–5.6]	4.9 [4.7–5.5]	
Iron, μmol/L	*n* = 26	14.1 [7.2–17.8]	8.1 [6.3–15.8]	
Receiving antibiotics	*n* = 33	6 (43)	6 (32)	
Receiving steroids	*n* = 33	0 (0)	0 (0)	
Receiving proton pump inhibitors	*n* = 33	7 (50)	7 (37)	

Acylated ghrelin, pg/mL		279.2 [126.1–580.4]^b^	126.4 [59.7–237.9]^c^	82.4 [56.3–130.4]
Unacylated ghrelin, pg/mL		101.0 [42.6–208.5]	84.5 [42.6–142.9]^c^	157.3 [79.3–261.0]
Acylated/unacylated ghrelin ratio		2.4 [1.0–4.3]^b^	1.4 [0.8–2.9]^c^	0.6 [0.4–0.8]

*IQR, interquartile range; PN, parenteral nutrition; SDS, standard deviation score.*

*^a^Dutch healthy control subjects from a study from Kuppens et al. (15), using the same method for AG and UAG measurement.*

*^b^Levels in patients with short bowel syndrome significantly differed from healthy control subjects (for AG: p = 0.002; for AG/UAG: p = 0.001), analyzed with one-sample Wilcoxon signed rank test.*

*^c^Levels in patients with functional IF significantly differed from healthy control subjects (for AG: p = 0.023; for UAG: p = 0.015; for AG/UAG: p < 0.001), analyzed with one-sample Wilcoxon signed rank test.*

#### Acylated and Unacylated Ghrelin Levels

We included 1 or 2 samples per patient, resulting in a total of 27 AG and UAG measurements. Fifty-seven percent of the samples was drawn after 4 h of fasting; for the remaining samples fasting was not feasible due to practical issues such as tube feeding times. Median baseline AG and UAG levels with corresponding AG/UAG ratio were 279.2 pg/mL (IQR 126.1–580.4), 101.0 pg/mL (IQR 42.6–208.5), and 2.4 (IQR 1.0–4.3), respectively. Median AG level and AG/UAG ratio were significantly higher than in the reference population (*p* = 0.002 and *p* = 0.001, respectively); median UAG level did not significantly differ (Table 1) (15). No differences in AG and UAG levels were seen between patients with and without antibiotics, patients with and without proton pump inhibitor use, or between 4 h fasted and non-fasted patients.

For patients with follow-up AG and UAG measurements (*n* = 11), median duration between the first and last AG and UAG measurement was 2.0 years (IQR 0.5–2.1). From the first to the last measurement, median AG level did not change significantly [211.3 pg/mL (IQR 108.2–605.3) to 158.0 pg/mL (IQR 79.9–674.8), *p* = 0.657], neither did PN-dependency [53.5% (IQR 40.3–100) to 57.0% (IQR 38.6–100), *p* = 0.779]. UAG level did significantly decrease [98.6 pg/mL (IQR 45.7–262.0) to 26.4 pg/mL (IQR 8.6–140.4), *p* = 0.021].

#### Associations Between Ghrelin and Nutritional Intake

**FIGURE 1:**
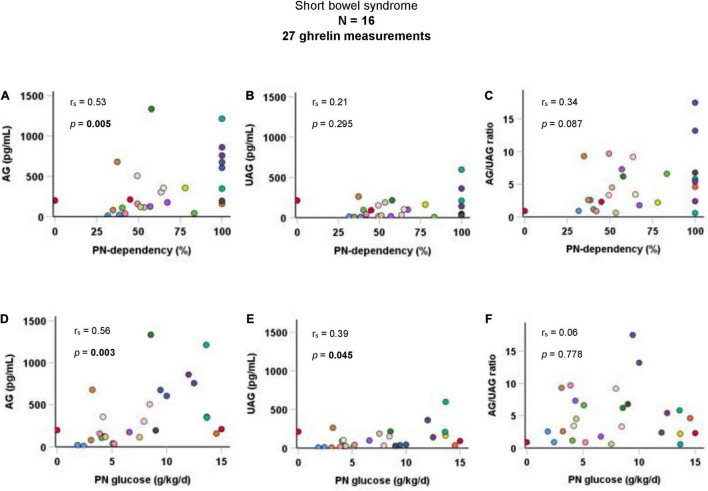
Correlations of AG, UAG levels, and AG/UAG ratio with **(A–C)** PN-dependency, and **(D–F)** parenteral glucose intake in g/kg body weight per day in patients with short bowel syndrome. AG, acylated ghrelin; PN, parenteral nutrition; UAG, unacylated ghrelin. Every dot represents one ghrelin measurement. Dots in the same color are from the same patient. Correlations between variables were quantified using Spearman’s rho (r_s_).

Taking all 27 AG and UAG measurements together, PN-dependency was significantly correlated with AG (r_s_ = 0.5, *p* = 0.005), but not with UAG or AG/UAG ratio. Additionally, there was a significant correlation between parenteral glucose intake (g/kg body weight per day) and both AG (r_s_ = 0.6, *p* = 0.003) and UAG (r_s_ = 0.4, *p* = 0.045). Correlations are visualized in Figure 1. Age inversely correlated with both AG (r_s_ = −0.6, *p* < 0.001) and UAG (r_s_ = −0.6, *p* = 0.003), but also with PN-dependency (r_s_ = −0.4, *p* = 0.027) and parenteral glucose intake (r_s_ = −0.7, *p* < 0.001). There was no correlation between fasting duration and any of the ghrelin parameters, PN-dependency or parenteral glucose intake.

### Functional Intestinal Failure

#### Patient Characteristics

We included 20 patients with functional IF [median age 7.3 years (IQR 4.1–13.8), 11 females]. The most common underlying disease was pediatric intestinal pseudo-obstruction (*n* = 9), followed by microvillus inclusion disease (*n* = 3). None of the patients underwent gastric or duodenal resection. At the time of the first AG and UAG measurement, included patients were dependent on PN for a median of 6.6 years (IQR 2.4–13.2) for 100% of their nutritional intake (IQR 77.4–100). Patient characteristics are shown in Table 1.

#### Acylated and Unacylated Ghrelin Levels

We included 1–2 samples per patient, resulting in a total of 37 AG and UAG measurements. Sixty-three percent of the samples was drawn after 4 h of fasting; for the remaining samples fasting was not feasible due to practical issues such as tube feeding times. Median baseline AG and UAG levels with corresponding AG/UAG ratio were 126.4 pg/mL (IQR 59.7–237.9), 84.5 pg/mL (IQR 42.6–142.9) and 1.4 (IQR 0.8–2.9), respectively. Median AG level and AG/UAG ratio were significantly higher than in the reference population (*p* = 0.023 and *p* < 0.001, respectively) and median UAG level was significantly lower than in the reference population (*p* = 0.015) (Table 1) (15). No differences in AG and UAG levels were seen between patients with and without antibiotics, patients with and without proton pump inhibitor use, or between 4 h fasted and non-fasted patients.

For patients with follow-up AG and UAG measurements (*n* = 17), median duration between the first and last AG and UAG measurement was 1.2 years (IQR 0.8–1.5). From the first to the last measurement, no significant changes were seen in AG level [189.6 pg/mL (IQR 105.2–262.3) to 252.5 pg/mL (IQR 155.9–369.8), *p* = 0.344], neither in UAG level [95.6 (IQR 43.6–164.0) to 73.6 (IQR 55.6–123.5), *p* = 0.492]. PN-dependency also did not change [100% (IQR 77.9–100) to 100% (57.3–100), *p* = 0.069].

#### Associations Between Ghrelin and Nutritional Intake

**FIGURE 2:**
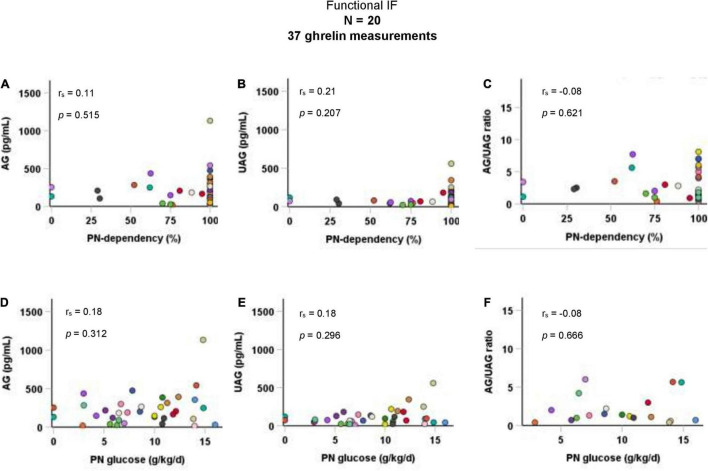
Correlations of AG, UAG levels, and AG/UAG ratio with **(A–C)** PN-dependency, and **(D–F)** parenteral glucose intake in g/kg body weight per day in patients with functional IF. AG, acylated ghrelin; IF, intestinal failure; PN, parenteral nutrition; UAG, unacylated ghrelin. Every dot represents one ghrelin measurement. Dots in the same color are from the same patient. Correlations between variables were quantified using Spearman’s rho (r_s_).

Taking all 37 AG and UAG measurements together, there were no significant correlations between PN-dependency and AG, UAG, or AG/UAG ratio in children with functional IF. Parenteral glucose intake (g/kg body weight per day) was also not correlated with any of the ghrelin parameters (Figure 2). Age was inversely correlated with parenteral glucose intake (r_s_ = −0.5, *p* = 0.001), but not with ghrelin parameters. There was a positive correlation between fasting duration and UAG levels (r_s_ = 0.4, *p* = 0.023).

### Differences Between Short Bowel Syndrome and Functional Intestinal Failure

**FIGURE 3:**
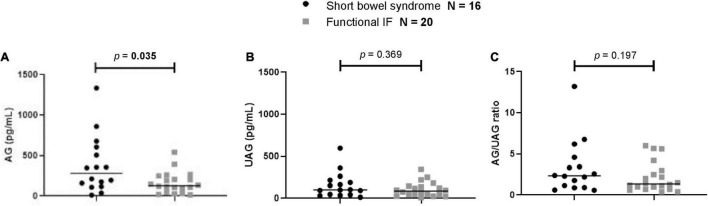
Comparison of first measurement of **(A)** AG, **(B)** UAG levels, and **(C)** AG/UAG ratios between patients with functional IF (gray) and patients with short bowel syndrome (black). AG, acylated ghrelin; IF, intestinal failure; UAG, unacylated ghrelin. Every (black or gray) symbol represents one ghrelin measurement in one patient. The median is shown as a horizontal line in front of the symbols. Patient groups were compared using Mann–Whitney U tests.

At baseline, patients with SBS had a significantly higher median AG level than patients with functional IF (*p* = 0.035). No significant differences between patients with SBS and functional IF were found for UAG levels (*p* = 0.369) or AG/UAG ratios (*p* = 0.197) (Figure 3).

## Discussion

In this multicenter study, we aimed to gain insight into the underlying mechanisms of intolerance to EN in children with IF receiving long-term PN by exploring the role of ghrelin. We found increased levels of AG in children with IF compared with healthy peers. Children with SBS had higher levels of AG than children with functional IF. Moreover, PN-dependency and parenteral glucose intake were positively correlated with AG levels in children with SBS.

This is the first study to assess ghrelin levels in children with IF receiving long-term PN. Currently, there are no standardized reference values for AG and UAG levels in children available. Therefore, we compared AG and UAG levels with healthy children from another study in the same age range as the children from our study cohort, using the same methods for AG and UAG measurement as the present study (15). In our patients with SBS, median AG level was more than three times higher. The higher AG levels led to an elevated AG/UAG ratio, compared with healthy children. Starvation of the gut may explain these findings of higher AG levels in children with SBS compared with healthy children. In addition, there are a few studies in adults with SBS concerning ghrelin levels. In a study including 24 adult patients with SBS, decreased plasma total ghrelin levels were seen (14), which according to the authors is explained by a significant reduction in tissue mass (of the intestine) that is able to secrete ghrelin. However, the stomach, which is the main site of ghrelin production, was preserved in all patients. The decreased ghrelin levels are not in line with our finding of higher AG levels in children with SBS (who also have a reduction of intestinal tissue mass) compared with children with functional IF (who have their whole small intestine *in situ*). Of note is that a different method for ghrelin measurement was used (without distinction of AG and UAG), making comparison difficult (14). In another study including nine adult patients with SBS, increased active ghrelin plasma levels were demonstrated (12). The authors also concluded that postprandial inhibition of ghrelin levels was delayed, suggesting that a hunger signal persisted after initiation of a meal. Sometimes, AG is referred to as “active” ghrelin, but also in this study, a different method for ghrelin assessment was used, making a direct comparison complicated.

A patient population with nutritional challenges in which AG and UAG levels have been studied more often are children with Prader–Willi syndrome. Prader–Willi syndrome is characterized by poor feeding, failure to thrive, and muscular hypotonia in infancy. In these infants, elevated levels of UAG with low AG/UAG ratios were seen, suggesting that UAG is associated with poor feeding (20, 21). In older children with Prader–Willi syndrome with hyperphagia and impaired satiety, increased levels of AG (129.1 pg/mL) with elevated AG/UAG ratios were observed (15). Both children with Prader–Willi syndrome and children with IF (SBS and functional IF) are known to have an altered body composition with increased fat mass and decreased fat-free mass (22, 23). The lipogenic effect of AG may explain the similarities between the patient groups (24).

We found PN-dependency to be positively correlated with AG levels in patients with SBS. This supports the concept of the previously mentioned starvation of the gut. Starvation itself leads directly to raised AG levels which, in mouse models, has been shown to protect against hypoglycemia by increasing growth hormone levels and subsequent development of insulin resistance (7, 12, 13). We speculate that although ghrelin levels are increased in children with SBS due to gut starvation, these increased levels are not able to maintain euglycemia in these patients who therefore require increased parenteral glucose intake. The link between ghrelin and glucose metabolism may be disrupted in children with SBS. Disorders of glucose metabolism are a possible complication of PN, although their mechanisms are not clear. A recent preliminary study suggests that patients receiving PN are insulin resistant and hyperinsulinemic, despite good control of glycemic state (25). Another study shows that PN suppresses glucose induced insulin secretion by the pancreas (26). Based on these findings, the strong association between AG and glucose infusion rate in children with SBS suggests that AG levels may also indicate the level of glucoregulatory function in these patients, although further studies are needed to confirm this relationship. It would be interesting to evaluate glucose-insulin homeostasis and levels of other hormones such as glucagon-like peptide 1 (GLP-1), peptide YY (PYY), motilin, leptin, adiponectin, and growth hormone.

Patients with functional IF also seem to have raised levels of AG compared with healthy children. However, we cannot explain why children with SBS have significantly higher levels of AG than children with functional IF. Children with SBS had lower PN-dependency than children with functional IF, but not necessarily more enteral intake. Children with pseudo-obstruction, for example, often have a jejunostomy which they empty after eating, resulting in intake that does not count as energy intake since it is not absorbed in the intestine. Possibly, considering that the fundus of the stomach is the primary site of ghrelin secretion, there might be a difference in gastric function between patients with SBS and functional IF because of a distended stomach in functional IF. We found no correlation between AG or UAG levels and PN-dependency in children with functional IF. This may be because the majority of the samples (62%) were from patients with 100% PN-dependency.

A major strength of the current study is that it is the only study of its kind in children with IF which suggests a physiological role for ghrelin in the response to PN in pediatric IF. Another strength is the method of ghrelin assessment. Where previous studies used radioimmunoassay for total ghrelin measurement (14, 27), we measured both AG and UAG. This was done with double-antibody sandwich ELISA to prevent detection of inactive fragments which lead to overestimation of peptide levels. Also, we stabilized the samples. Inevitably, there are also limitations. In 50 out of 109 ghrelin measurements, patients were receiving 100% of their nutritional intake from PN. Possibly, the correlation between PN-dependency and AG level is even stronger in a population of patients with more variation in PN-dependency. Moreover, not all patients could be fasted before sampling (nearly 50%), which could have affected (lowered) the average level of AG measured. We were also not able to take into account the diurnal pattern of ghrelin release (28). The small sample size of our study did not allow us to correct for multiple factors that may have influenced ghrelin levels. We were not able to remove these influencing factors either for ethical reasons (such as medication use and overnight infusion of PN) or because these were not modifiable influencing factors (such as sex and age). For example, age was inversely correlated with both AG/UAG levels and PN-dependency/parenteral glucose intake. We do not know how much of the correlation between AG/UAG levels and PN-dependency/parenteral glucose intake is explained by age.

## Conclusion

In this exploratory descriptive study, children with IF had raised AG levels compared with an age-matched control group, which might be related to starvation of the gut. The positive correlation of AG with PN-dependency and glucose infusion rate in patients with SBS suggests an association with altered glucoregulatory function in these patients, although further studies are needed to explore this concept.

## Data Availability Statement

The raw data supporting the conclusions of this article will be made available by the authors, without undue reservation.

## Ethics Statement

The studies involving human participants were reviewed and approved by the local research ethical committees from the Erasmus MC University Medical Center Rotterdam and the AMC Amsterdam University Medical Center (MEC 2015-002, Dutch Trial Register NL5892, https://www.trialregister.nl/trial/5892). Written informed consent to participate in this study was provided by the participants’ legal guardian/next of kin.

## Author Contributions

LV, PD, EN, JV, RW, ER, MT, JH, and BK contributed to study concept and design. LV, PD, EN, MH, JV, SN, MT, JH, and BK contributed to acquisition, analysis, and interpretation of data. LV, PD, EN, and BK contributed to drafting of the manuscript. All authors critically revised the manuscript for important intellectual content and read and approved the final version of the manuscript.

## Conflict of Interest

The authors declare that the research was conducted in the absence of any commercial or financial relationships that could be construed as a potential conflict of interest.

## Publisher’s Note

All claims expressed in this article are solely those of the authors and do not necessarily represent those of their affiliated organizations, or those of the publisher, the editors and the reviewers. Any product that may be evaluated in this article, or claim that may be made by its manufacturer, is not guaranteed or endorsed by the publisher.

## Footnotes

1
https://growthanalyser.org/software/growth-analyser-rct/

2
http://www.graphpad.com
